# Spinal arthritis in cane toads across the Australian landscape

**DOI:** 10.1038/s41598-018-30099-0

**Published:** 2018-08-20

**Authors:** Deborah S. Bower, Kiyomi Yasumiba, Daryl R. Trumbo, Ross A. Alford, Lin Schwarzkopf

**Affiliations:** 1 College of Science and Engineering, James Cook University, Townsville, 4811 Queensland Australia; 2 Washington State University, School of Biological Sciences, Pullman, WA 99164 United States of America; 3 Present Address: School of Environmental and Rural Science, University of New England, Armidale, 2351 New South Wales Australia

## Abstract

Loss of fitness can be a consequence of selection for rapid dispersal ability in invasive species. Increased prevalence of spinal arthritis may occur in cane toad populations at the invasion front as a cost of increased invasiveness, but our knowledge of the ecological drivers of this condition is lacking. We aimed to determine the factors explaining the prevalence of spinal arthritis in populations across the Australian landscape. We studied populations across a gradient of invasion histories. We collected 2415 toads over five years and determined the presence and size of spondylosis for each individual. We examined the effect of host size, leg length and invasion history on the prevalence of spondylosis. Host size was a significant predictor of spondylosis across populations. Contrary to our expectation, the overall prevalence of spondylosis was not positively related to invasion history and did not correlate with toad relative leg length. Rather than invasion age, the latitude at which populations were sampled provided an alternate explanation for the prevalence of spondylosis in cane toad populations and suggested that the incidence of this condition did not increase as a physiological cost of invasion, but is instead related to physical variables, such as climate.

## Introduction

Strong selection pressures on invading species enable rapid evolution of traits that increase invasiveness in novel habitats. Traits that improve the capacity of a species to invade, however, can incur costs to other aspects of its biology. For example, invasive plant species that thrive in benign habitats may pay a cost in the form of reduced ability to persist in more stressful environments^1^. Mounting evidence suggests that selection on species at invasion fronts can drastically change behaviour and physiology in invading populations, potentially leading to fitness trade-offs. For example, in birds, increased aggression and male size typically increase invasiveness at a cost to parental care and overall breeding success^2^. Determining the costs of invasion is an important aspect of understanding evolution at invasion fronts, a requirement to accurately predict and manage future invasions.

Cane toads (*Rhinella marina*) have been spreading across Australia since the late 1930s, evolving novel morphologies and behaviours that have accelerated their rate of dispersal^3^. These traits include faster individual growth rates, increased relative leg length and higher endurance^4,5^ in toads at the invasion front. Costs of these traits may include reduced immune function^6,7^ and increased incidence of spinal arthritis, which is posited to be caused by an interaction between degenerating joints and infection with a Brucella [*Ochrobactrum*] species (mean prevalence approximately 7.5%)^8–10^. In a previous study, the incidence of spinal arthritis was positively associated with large individual toads and populations with longer legs^8^.

Although spinal arthritis may be a consequential cost of increased invasion ability^8,9^, the mechanism explaining the incidence of spinal arthritis in various populations remains unclear. Despite high rates of spinal arthritis in toad populations at the front, studies of immunocompetence suggest that toads in these populations have the capacity, similar to that of toads in long established populations^6^, to inhibit bacterial infections (i.e., *Brucella spp.*). If spinal arthritis is indeed a cost of invasion, long-established toad populations, (e.g., near Townsville) with lower endurance^5^ and reduced movement^11^ should have lower prevalence of spinal arthritis than populations at the invasion front.

We studied toads across a gradient of invasion histories and hypothesized that: (1) larger toads would have a higher prevalence of spinal arthritis, (2) the overall prevalence of spinal arthritis would be higher in recently invaded areas, and, (3) toads with longer legs should have a disproportionately high prevalence of spinal arthritis relative to toads with shorter legs.

Here we demonstrate that, contrary to our expectations, spondylosis cannot be predicted by invasion history or leg length in toad populations across the Australian landscape but rather, the size of an individual and the latitude of occurrence of the population are the most important predictors of spinal condition.

## Methods

**Figure 1:**
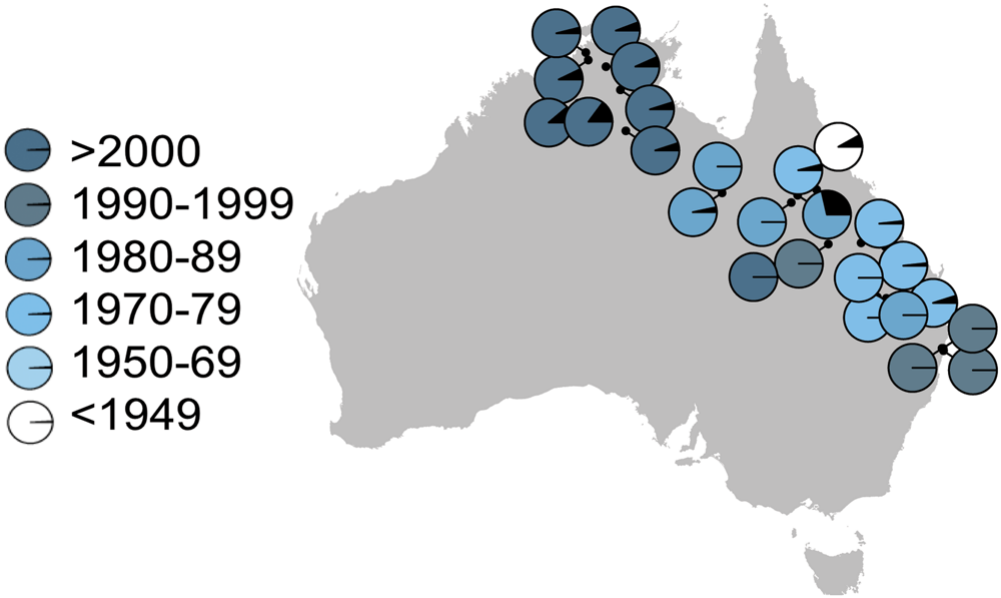
The proportion of spinal arthritis in toad sampling locations was greater in northern latitudes and was not affected by year of population establishment. Generated with R Studio 1.0.143 (https://www.R-project.org/) using package ‘mapplots’ and ‘marmap’.

We collected 1622 individual toads from the campus of James Cook University in Townsville, Queensland, Australia (-19.3329°S 146.7575^o^E) over five years (Supplementary Table S1) and an additional 727 toads from locations in the Northern Territory, Western and Southern Queensland and New South Wales in 2010 and 2011 (Fig. 1). Toads were captured by visual encounter and hand collection at night, or in traps^12,13^. They were euthanised by an overdose of buffered MS222, and preserved by freezing before dissection. During dissection, we used Vernier callipers to measure body size (snout-urostyle length) and the length and width of any obvious (>5.5 mm growth) spinal abnormality (which typically presented as bulbous growths around the vertebrae) to the nearest 0.1 mm. We determined sex by direct examination of the gonads. Research was approved by James Cook University’s animal ethics committee (A1617) and all methods were performed in accordance with relevant guidelines and regulations.

### Statistical Analysis

To determine whether there were sex or age effects on the prevalence (proportion of individuals showing signs of disease) of spinal arthritis, we used logistic regression with presence or absence of spinal arthritis as the binary response variable, and sex and stage (male, female, juvenile) as predictive factors. Invasion history was estimated from records from the Queensland museum, from Phillips and Shine^14^, and from Urban, *et al*.^15^. To determine the influence of invasion history, latitude and body size on the prevalence of spinal arthritis, we used a logistic regression with spinal arthritis as the binary response variable, latitude and year of establishment as additive factors and then year of establishment and snout-urostyle length as interactive predictive factors. To determine whether the presence or absence of spinal arthritis was influenced by relative leg length we first removed n = 182 individuals smaller than the smallest toad with spinal arthritis from the data set. We then used residuals from a correlation between log_10_ snout-urostyle length and log_10_ tibia length as predictor variables in a logistic regression with presence or absence of spinal arthritis as the response variable. Furthermore, we explored the interactions we observed between snout-urostyle and tibia length by examining interactions in a general linear model with spinal arthritis (binary) as the response variable, and log_10_ snout-urostyle length, and log_10_ tibia length as a predictive variable. To determine if there was a maximum threshold in the relationship between toad size and the size of the arthritis, i.e., if the inflamed area increased with toad size, we considered only toads with symptoms of arthritis. We subtracted the predicted width of healthy toad spines from the widest part of the spinal arthritis. Predicted widths were determined from a measured subsample of toads with healthy vertebrae. We performed a quantile regression with snout-urostyle length as the predictor variable and corrected width of spinal arthritis as the response variable, using the package ‘quantreg’^16^. All analyses were completed in R Studio^17^, map figures were generated using the ‘mapplots’ and ‘marmap’ package^18,19^, histograms were generated with ggplot2^20^.

### Data Accessibility

Data supporting our results is archived in James Cook University’s ‘Tropical Data Hub’ https://research.jcu.edu.au/researchdata/default/home.

## Results

Prevalence of spinal arthritis increased as latitudes became more tropical (Z = 4.29, P < 0.0001). Many populations in the southern part of the cane toad’s range lacked arthritic individuals, and some long-established populations (i.e., Hughenden and Townsville) had a high prevalence of spinal arthritis (Fig. 1). Statistically, year of establishment had a negative effect on the presence of spinal arthritis because many southern sites established in the 1980s and 1990s had a low prevalence of spinal arthritis (Z = −2.84, P = 0.004; Fig. 1).

**Figure 2:**
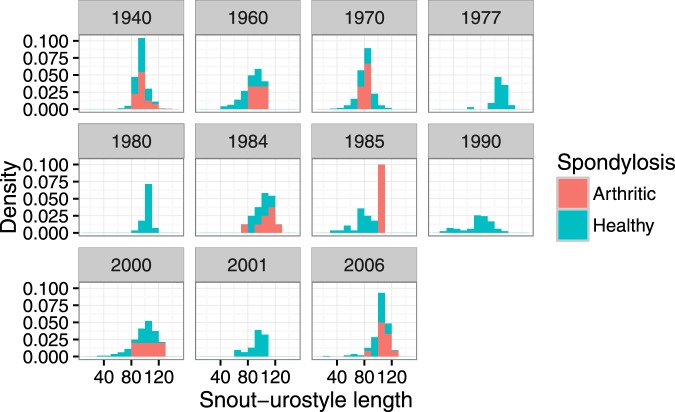
Size distribution of toad populations in samples from populations with different invasion histories.

Prevalence of spinal arthritis was significantly higher in females (10%) than in males (6%; Z = −5.74, P < 0.0001), or juveniles (<1%; Z = −5.98, P < 0.0001). Snout-urostyle length was positively correlated with spinal arthritis, with more arthritis in large toads (Z = 5.4, P < 0.0001) and there was a significant interaction between year of establishment and snout-urostyle length (Z = 2.88 P = 0.003) because even large toads did not have spinal arthritis in some populations (Fig. 2).

**Figure 3:**
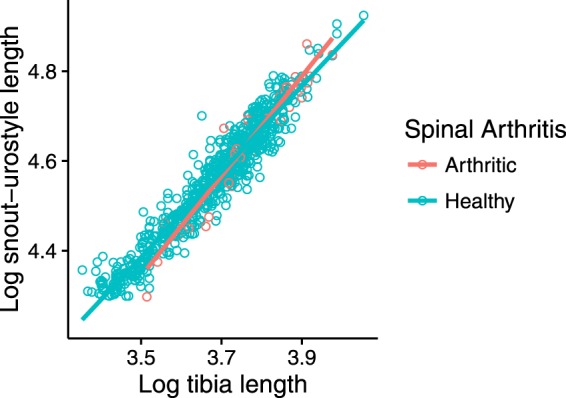
The relationship of leg length to body size was not significantly different in toads with and without spinal arthritis.

The relative leg lengths of toads with and without spinal arthritis did not differ significantly (Z = 0.10, P = 0.92). There was no significant interaction between the effects of tibia length and snout-vent length on the occurrence of spinal arthritis (Z = −0.637, P = 0.524; Fig. 3).

**Figure 4:**
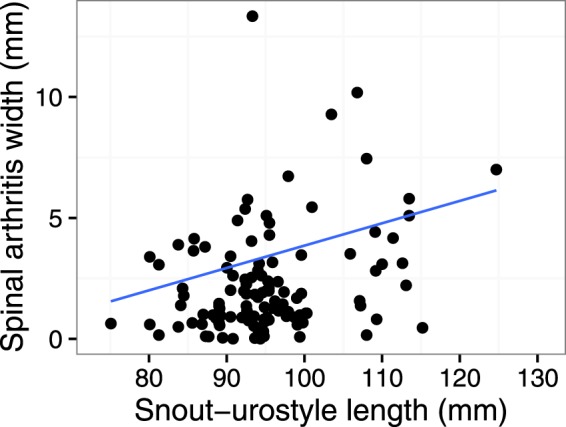
The width of spinal inflammation was positively correlated with snout-urostyle length of toads. Variability in the width of spinal inflammation also increased with size of toad. Blue line indicates the 80^th^ quantile threshold of the relationship of width of spondylosis to body size, indicating that as toads get larger, the affected area is also significantly larger, suggesting the condition worsens with age.

Snout-urostyle length of toads was positively correlated with the width of the spinal arthritis (tau = 0.8, t = 2.47, P = 0.01; Fig. 4), and the variance in width of spinal arthritis increased as snout-urostyle increased. The width of spinal arthritis observed (80^th^ percentile) increased at 0.09 ± 0.04 mm^2^ for every 1 mm increase in snout-urostyle length.

## Discussion

Latitude was a significant predictor of the prevalence of spinal arthritis among populations of cane toads in the Australian landscape. Within populations, toad size was an important determinant of spinal arthritis, and other factors we examined, such as relative leg length, were not significant predictors of spinal arthritis across the Australian landscape. Together, these results suggest that differences among populations in the prevalence of spinal arthritis may not reflect a cost, in the form of reduced immunity, of adaptation to increased invasiveness^3^ but may be better explained by differences in size structure and variation in the environment to which populations are exposed.

Many factors, including temperature and humidity, vary with latitude, and there is no available information on geographic variation in the prevalence of the putative cause of spinal arthritis (*Brucella spp*.) in relation to these variables. Possibly, warmer or wetter environments, or both, are conducive to rapid aging or susceptibility of toads to spinal deformities. Toads move more when it is warmer and wetter^21^, so spinal arthritis may be more common when toads move around more in their lifetime but this does not appear to be in relation to overall population invasion speed.

The width of spinal inflammation in individuals in our populations increased positively with toad size, such that the largest toads had the largest lesions, whereas smaller toads did not experience noticeable lesions. Larger lesions are likely to reflect the amount of time these large animals have been affected, as larger toads are likely to be older^22^. Living longer may also be associated with degeneration of joints, or may increase the time available to contract *Brucella* spp. and the positive relationship between the size of toads and the width of spinal arthritis suggests that larger toads, which are likely to also be older, have had spinal arthritis for longer.

Little is known about the epidemiology of *Brucella* spp. in relation to cane toad hosts, and we did not test for its presence in the lesions in our study. If *Brucella* spp. are the primary determinant of spinal arthritis, then density dependence, climatic suitability and genetic immunity may all play a large role in explaining population prevalence of infection^23^. However, Koch’s postulates have not been satisfied for the association of *Brucella* spp. with spinal arthritis in cane toads, and not all toads experiencing spinal arthritis have tested positive for *Brucella* spp. In toads, this bacterium may act similarly to its behaviour in human hosts, where it is innocuous in healthy individuals but pathogenic in immunocompromised individuals^24^. If joint degeneration from aging is a prerequisite for opportunistic bacterial infection, age (and likely size structure) in the population will be an important determinant of infection prevalence^22^.

Toads at the invasion front continue to evolve morphologies and behaviours that increase their invasive capacity^3^. Investment of energy in invasion may come at a cost, such as lowered immune function^7^, but the nuances of these mechanisms remain unclear^6^. Toads in populations at the invasion front have behavioural and morphological traits that enable them to disperse further than toads in long-established populations^11^ and yet the prevalence of spinal arthritis did not follow a clear trend in relation to invasion history, nor was it correlated with individual leg length. Our data was collected from a range of establishment histories and yet, populations closest to the invasion front had varying prevalence of spinal arthritis (from 4% in Victoria River to 17% in Timber creek) that overlapped the range of values sampled in populations at the front in 2005-2007, closer to the time of invasion (>14% in individuals larger than 110 cm Brown, *et al*.^8^). It, therefore, does not appear that increased prevalence of spinal arthritis is a cost of adaptation to facilitate invasion. Instead mechanisms that address the interplay between environmental conditions, growth of toads and size are required to unravel the mechanisms causing spinal arthritis.

## Electronic supplementary material

S1
