# Supplementary material for: Spinal arthritis in cane toads across the Australian landscape
Source: Sci Rep. 2018 Aug 20;8:12458. doi: 10.1038/s41598-018-30099-0 (PMC6102202; doi:10.1038/s41598-018-30099-0)
Supplement: Supplementary file 1 — S1 [file 41598_2018_30099_MOESM1_ESM.pdf]

Spinal arthritis in cane toads across the Australian landscape

Deborah S. Bower[1,2*], Kiyomi Yasumiba[1], Daryl R. Trumbo[3], Ross A. Alford[1], Lin Schwarzkopf[1]

[1]School of Science and Engineering, James Cook University, Townsville, 4811, Queensland, Australia
[2]Present address: School of Environmental and Rural Science, University of New England, Armidale, 2351, New South Wales, Australia
[3]Washington State University; School of Biological Sciences; Pullman, WA 99164, United States of America

Supplementary material 1. Site location information and sample size for spinal arthritis data 286 collection

Site	Latitude	Longitude	Proportion of spinal arthritis in sample	Year group	Sample size
Adelaide River	-13.23875	131.10702	7.69	2000	26
Boonah	-27.99304	152.68203	5.13	1960	39
Brooms Head	-29.6013	153.33015	0	1990	50
Burk & Wills	-19.22727	140.34719	0	1980	12
Darwin River Dam	-12.82038	130.96219	4	2000	25
Duaringa	-23.7183	149.66921	1.72	1970	58
Dullacca	-26.64402	149.75827	0	1970	20
Emerald	-23.53025	148.16624	1.72	1960	58
Hughenden	-20.84212	144.20223	40	1980	20
Jabiru	-12.67143	132.83982	6.06	2000	33
Jericho	-23.60191	146.12563	0	1990	31
Kakadu	-13.60617	132.21847	7.14	2000	14
Mataranka	-14.92207	133.13333	5.13	2000	39
Micalo Island	-29.44159	153.31261	0	1990	28
Mount Isa	-20.70824	139.49384	3.45	1980	29
Newcastle Waters	-17.23286	133.46547	5.26	2000	19
Pentland	-20.52703	145.39607	4.08	1970	49
Stamford	-21.261	143.81797	0	1980	2
Stonehenge	-24.35421	143.28686	0	2000	31
Timber Creek	-15.66351	130.48095	12.5	2000	32
Toowoomba	-27.58989	151.9716	0	1980	28
Townsend	-29.46421	153.23614	0	1990	47
Townsville	-19.20771	146.7624	8.75	1940	1622
Victoria River	-15.61462	131.12966	17.39	2000	23
Wullumbilla	-26.58026	149.18056	0	1970	14
